# Neonatal cardiorespiratory failure caused by splenic hemorrhages: a case report on a fatal and a rescued case

**DOI:** 10.1186/s12887-024-05002-8

**Published:** 2024-09-05

**Authors:** Nadieh A. Jansen, Anne T M Dittrich, Manouk Backes, Benno Kusters, Willemijn M Klein, Tim Hundscheid

**Affiliations:** 1 Department of Pediatrics, Division of Neonatology, Radboud University Medical Center, Amalia Children’s Hospital, Nijmegen, The Netherlands; 2 Department of Pediatrics, Radboud University Medical Center Radboud University Medical Center, Amalia Children’s Hospital, Nijmegen, The Netherlands; 3 Department of Pediatric Surgery, Radboud University Medical Center Radboud University Medical Center, Amalia Children’s Hospital, Nijmegen, The Netherlands; 4 Department of Pathology, Radboud University Medical Center, Nijmegen, The Netherlands; 5 Department of Medical Imaging, Radboud University Medical Center, Nijmegen, The Netherlands

**Keywords:** Splenic hemorrhage, Splenic bleeding, Neonate, Case report, Birth trauma

## Abstract

Two cases of neonatal splenic hemorrhage with acute cardiorespiratory failure are described in this report. The first case involves a full-term neonate who was found unresponsive without any witnesses and could not be successfully resuscitated. A postmortem diagnosis revealed a splenic hemorrhage. Second case is an extremely premature neonate who experienced a witnessed cardiovascular collapse on the 14th day of life. Rapid cardiovascular support was administered, resulting in a positive outcome. While splenic hemorrhage is commonly associated with traumatic events, these cases highlight the need of considering spontaneous splenic hemorrhages as a potential cause of acute neonatal compromise, even in the absence of birth-related trauma (e.g., asphyxia, prolonged labor, clavicle fractures, brachial plexus injuries). This report emphasizes the importance of including splenic hemorrhage timely in the differential diagnosis of neonatal cardiorespiratory instability, especially in the absence of more common diagnoses, and discusses the challenges associated with its recognition and treatment.

## Background

Cardiorespiratory failure in newborns can be regarded as a final common pathway, with a broad spectrum of potential underlying diagnoses. The primary considerations encompass sepsis, pulmonary hypertension, abdominal problems (e.g., obstruction, or necrotizing enterocolitis (NEC) in premature cases), blood loss (e.g., due to birth trauma or coagulation disorders, with cerebral bleeding being the most common), a (congenital) cardiac disorder or an inborn error of metabolism [1]. The diagnostic assessment involves the initial elimination and preemptive treatment of these most prevalent and/or life-threatening causes. However, when the cause of cardiorespiratory failure remains unknown after ruling out the most common causes, this article aims to demonstrate the importance of timely considering splenic hemorrhage in the differential diagnosis.

In children, a splenic hemorrhage most often results from blunt trauma [2, 3]. As the spleen is a highly vascular organ, there is a high risk of cardiorespiratory failure due to hypovolemic shock when a splenic hemorrhage occurs [4]. The clinical presentation of neonatal splenic hemorrhage is often nonspecific and may include pallor, anemia, abdominal distention, and eventually cardiorespiratory failure [1].

This report describes two cases of splenic hemorrhage in neonates as primary cause of cardiorespiratory failure, including a description of symptoms, diagnostics tests, management and outcome.

## Case description

### Case 1

This case involves a full-term male neonate who was born after an uncomplicated pregnancy and delivery, with a birth weight of 4400 g (p98). After clinical observation due to being large for gestational age with adequate glucose levels, he was discharged home. After intermittent non-alarming feeding difficulties within the first few days, on the third day, he was found unresponsive, pale, and floppy in his bed. The emergency services noticed a tense abdomen and asystole. Resuscitation was started according to current advanced life support guidelines, which involves the administration of fluid boluses (total of 20 ml/kg isotonic saline) and adrenaline (every 5 min 10 mcg/kg). As the cause of asystole remained unclear after forty minutes, resuscitation was discontinued as no further recovery was anticipated, after which the patient deceased.

**Fig. 1:**
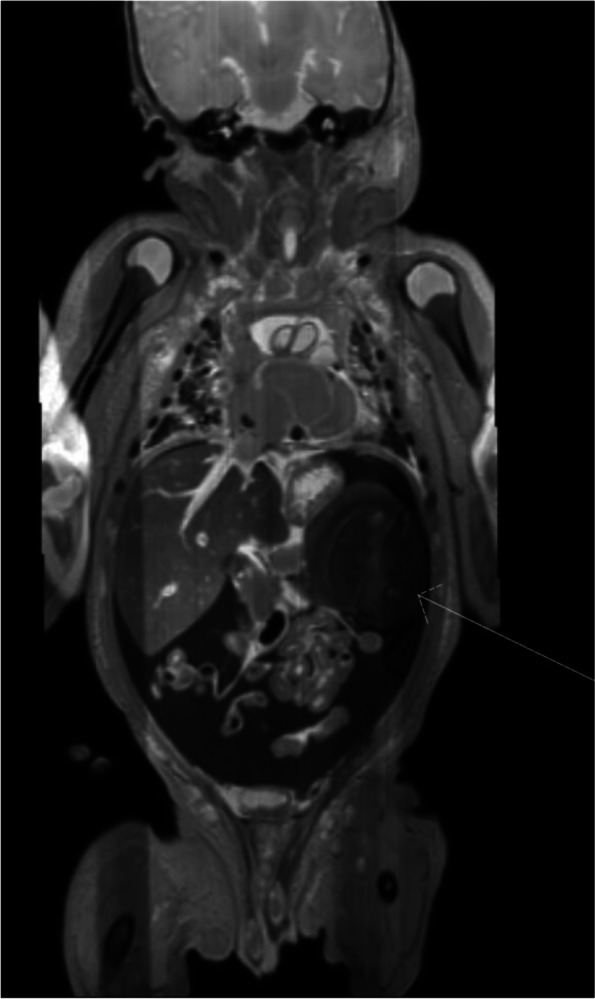
coronal T2 MR image of patient 1, performed postmortem. There is diffuse intra-abdominal blood. The arrow indicates the enlarged and inhomogeneous spleen. The image is indicative for a large splenic hemorrhage with capsular rupture

Post-mortem diagnostics were performed with parental consent to determine the cause of death. The most remarkable finding was a hemoglobin (Hb) level of 0,8 mmol/L. There were no signs of infection, with a C-reactive protein (CRP) of 9 mg/L, negative blood and spinal fluid cultures, as well as fecal and nasopharyngeal polymerase chain reaction tests. Magnetic resonance imaging (MRI) and computed tomography (CT) showed massive hemorrhage in and around the spleen (Fig. 1). Differential diagnostic causes included a spontaneous hematoma of the spleen or pathological mass (e.g., congenital hemangioma or another tumor). There were no fractures or (other) traumatic injuries visible, and no congenital malformations were observed.

At autopsy, the most remarkable findings included a very pale skin color, few corpse stains and a slightly bulging abdomen, all indicative of a large hemorrhage. Furthermore, macroscopic examination revealed enlargement of all organs, along with the presence of a partly friable and partly solid blood clot weighing 56 g, adhered to the spleen. A ruptured capsule on the spleen’s edge, with accompanying blood clot and fragments of spleen tissue indicated that the blood originated from the spleen. There was no evidence of trauma to the inner thoracic or abdominal walls, tumors, or vascular malformations, nor was there any sign of non-accidental injury. In the case history, there were no individuals in the family with coagulation problems, cardiovascular diseases at a young age, or acute cardiac death. Whole exome sequencing did not indicate any underlying hematological or metabolic disease. The probable cause of death was identified as a neonatal splenic hemorrhage, possibly after a birth trauma, considering the infant’s birth weight.

### Case 2

An extremely premature male infant, born after 24 + 5/7 weeks gestation with a birth weight of 650 g (p15), presented with acute cardiorespiratory failure at the postnatal age of two weeks. His medical history includes infant respiratory distress syndrome and apnea of prematurity. He received non-invasive respiratory support and caffeine. Two days before his clinical deterioration, he was extubated after the initiation of dexamethasone. Because of a suspected nosocomial infection, he received flucloxacillin and gentamicin until last evening. Antibiotics were discontinued after low inflammatory markers and negative blood culture. Echoencephalography showed an intraventricular hemorrhage grade 2 on the left side and periventricular echogenicity grade 1 on both sides of the brain.

His cardiorespiratory failure presented with a drop in his basal heart rate to 110 beats/minute without variation with numerous bradycardias. Clinical assessment showed a pale child, peripherally poorly circulated with increased work of breathing and tachypnea. Apart from a somewhat swollen abdomen, there were no indications of an abdominal problem. His arterial blood pressure measured 44/26 mmHg, with a mean of 35 mmHg. Despite the relatively low systolic pressure for his age, the mean blood pressure remained relatively normal. Arterial blood gas analysis showed a pH of 6.56, pCO_2_ 8.5 kPa, and remarkably, a hyperlactatemia of 13 mmol/L. The differential diagnoses included primary respiratory failure, primary cardiac failure, sepsis, abdominal focus, or blood loss.

**Fig. 2:**
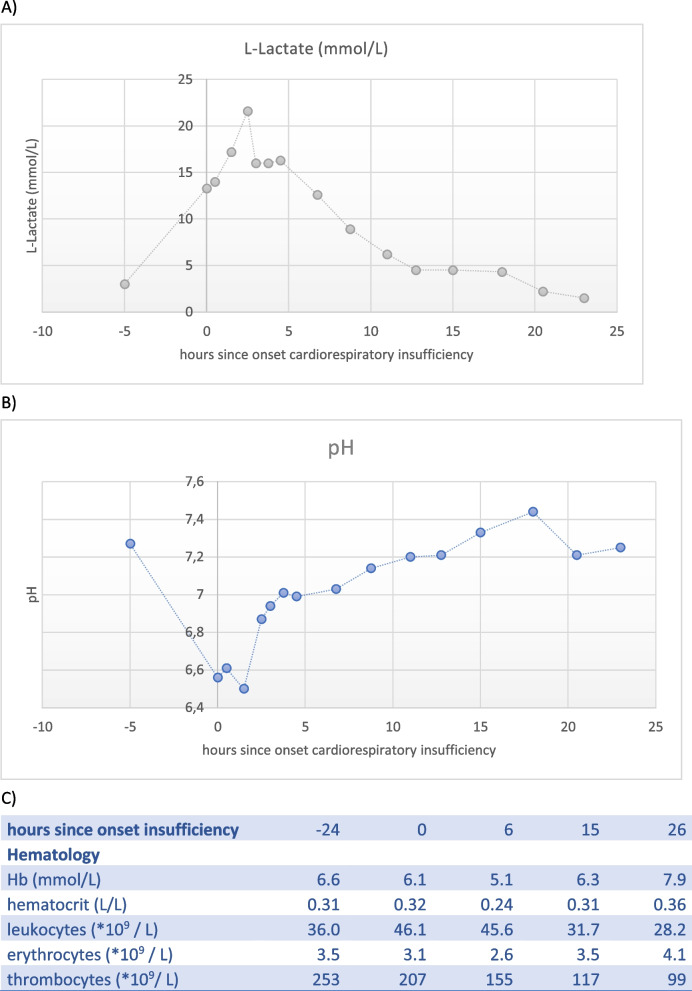
**A** L-lactate levels over time since the onset of cardiorespiratory insufficiency. **B** pH value over time since the onset of cardiorespiratory insufficiency. **C** Blood count values at the day of the cardiorespiratory insufficiency

He was intubated with low respiratory settings and Fraction of Inspired Oxygen (FiO_2_) of 0.4, making a primary respiratory problem less likely. Echocardiography showed a biventricular dysfunction with left ventricular dilatation, without primary cardiac pathology. The poor myocardial function was attributed to the metabolic dysregulation of the patient and was supported with dobutamine and a fluid bolus. Despite these interventions, the lactate levels rose to 17 mmol/L with a pH below the measurable threshold. Therefore, noradrenaline was administered, and dexamethasone was substituted for hydrocortisone. Sodium bicarbonate was administered to correct the metabolic acidosis. An overview of the most important blood results over 24 h is presented in Fig. 2.

Despite the recent discontinuation of gentamicin and flucloxacillin, the onset of severe cardiorespiratory failure and colonization with *Enterobacter cloacae* and *Serratia marcescens* prompted the initiation of high dose meropenem. Blood cultures did not grow a pathogen, which led to the discontinuation of antibiotics after four days.

Prior to the clinical deterioration, feeding was well tolerated, and defecation was normal. Despite some abdominal distension, no other features of NEC were seen on clinical examination by the pediatric surgeon and an abdominal X-ray revealed no abnormalities. Due to the significant clinical decline, enteral nutrition was halted and substituted for total parenteral feeding.

**Fig. 3:**
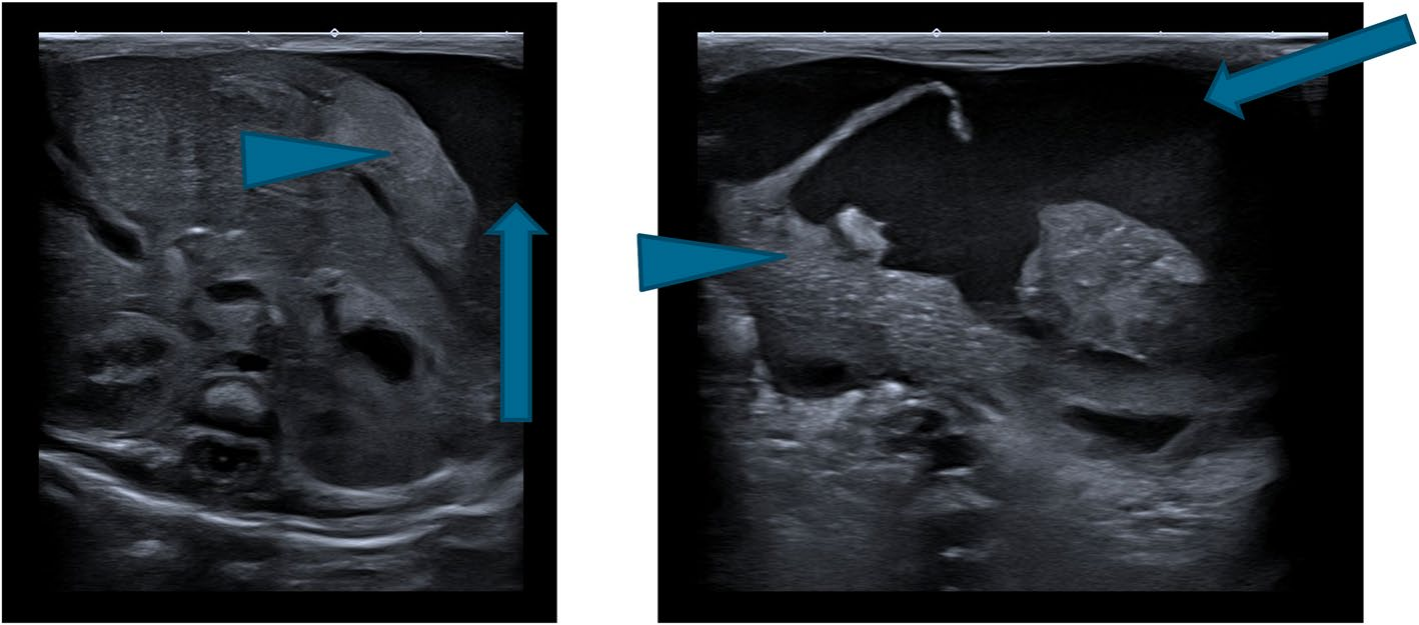
A. Transversal and B. sagittal ultrasound of case 2. The arrow indicates the fluid (hemorrhage) in the left upper abdomen. The arrowhead indicates the splenic parenchyma forming a border of the hemorrhage

As the hemoglobin rate dropped from 6.6 to 6 mmol/L in 24 h, an echoencephalography was performed, showing no signs of bleeding. In absence of a clear diagnosis, an abdominal ultrasound was performed by the pediatric radiologist. A capsulated fluid collection with an amorphous structure was found in the left upper abdomen. The structure caused compression on the spleen and stomach caudally, measuring 28 × 26 × 37 mm, estimated volume of 14 ml. Therefore, the ultrasound diagnosis was consistent with a subcapsular hemorrhage of the spleen (Fig. 3). As the hemorrhage of the spleen was confined to the subcapsular space and the symptoms were stabilizing, a surgical intervention was not indicated, and conservative management including repeated evaluation by the pediatric surgeon continued.

After the first fluid bolus (10 mL/kg), two erythrocyte transfusions (total 30 mL/kg), fresh frozen plasma (15 ml/kg), vitamin K 0.5 mg and circulatory support with dobutamine (maximum of 5.0 mcg/kg/min), hydrocortisone (5 mg/kg/day) and noradrenaline (maximum of 0.2 mcg/kg/min), cardiorespiratory stabilization was achieved, as depicted in Fig. 2. Within 24 h after the deterioration, dobutamine could be stopped and the noradrenaline was weaned. Four days later, the infant could be extubated to non-invasive respiratory support.

After hematological examination, no evidence was found for a coagulation disorder. On the acute moment there was prolongation of clotting times which was likely secondary to consumption during bleeding. Follow-up indicated normalization of clotting times.

There were no signs of organ dysfunction following the period of cardiorespiratory distress. Echoencephalographic follow up and MRI of the brain at term equivalent age showed no abnormalities. A follow-up abdominal ultrasound four weeks later showed a decreased volume of the splenic hemorrhage. Clinically, he recovered well from the incident and actual functions within the normal spectrum of an extremely preterm infant.

## Discussion

This report elucidates the potential implications of neonatal splenic hemorrhage leading to acute and severe cardiorespiratory failure.

Since the description of neonatal splenic rupture in 1967 [5], additional cases have been described and linked to birth trauma [4, 6]. The clinical presentation of neonatal splenic hemorrhage is often non-specific, with symptoms ranging from pallor, anemia, abdominal distention to cardiorespiratory failure [4]. A few case reports have even mentioned scrotal abnormalities as the sole symptom of a splenic rupture [7]. Most splenic hemorrhages occur within hours to several days after birth [4, 6, 8], supporting the primary hypothesis that birth trauma plays an important role in the occurrence of splenic hemorrhages. Increased intrathoracic pressure during birth may force the liver and spleen out of the diaphragmatic hollow. The pressure on the supporting ligament could potentially lead to splenic rupture by forming a subcapsular hematoma, followed by rupture of the capsule which leads to a hemodynamic instable patient [9]. This was possible the cause of the splenic hemorrhage in our first case considering the clinically notable swollen abdomen at presentation. In that case, the hematoma should have occurred during birth, and the capsule rupture three days later, after which his clinical condition deteriorated acutely.

Chang et al. presented a spectrum in the outcome after splenic hemorrhage. Notably the outcome ranged from mortality to complication-free survival irrespective of hemoglobin levels and traumatic or non-traumatic birth [4]. However, neonatal non-traumatic splenic hemorrhages are exceptionally rare and, consequently, they receive less consideration in the absence of anticipated birth trauma [4, 9]. Due to the low incidence of splenic rupture, systematic ultrasound screening of the spleen has not been conducted after traumatic or non-traumatic birth.

In our second case, the occurrence of a splenic capsular hemorrhage at the age of two weeks renders a traumatic hemorrhage due to a traumatic event at birth unlikely and emphasizes the lack of clarity regarding the precise pathophysiology of splenic hemorrhage [9].

Our second patient was an extremely premature neonate, a condition for which there are limited cases of splenic hemorrhage reported in the literature. One such case involved a neonate born after 27 weeks’ gestation [11]. In this case, clinical signs of hypovolemic shock, accompanied by metabolic acidosis and a decreased hemoglobin level 20 h postnatally, prompted urgent intervention. Surgical exploration revealed a subcapsular splenic hemorrhage, successfully managed without splenectomy, albeit with postoperative complications such as a subcapsular hematoma of the liver, acute renal failure, and disseminated intravascular coagulation [10].

In another case, a preterm neonate delivered at 32 weeks’ gestation, the challenges in managing cardiorespiratory failure attributed to splenic hemorrhage were evident. An emergency caesarean section was performed due to abnormal fetal heart rhythms, with a difficult extraction. The Apgar score was 3/3/3, with a venous pH of 7.31 and a cord hemoglobin level of 8.7 mmol/L The neonate required intubation at 10 min postpartum. At three hours of age, an acute episode of cardiorespiratory failure transpired, accompanied by disseminated intravascular coagulation and severe anemia (Hb 2.7 mmol/L). Nine hours later an abdominal ultrasound revealed a substantial subcapsular splenic hematoma with associated hemoperitoneum. Despite judicious fluid management, the prognosis remained unfavorable due to multi-visceral failure, hepatocellular insufficiency, and pulmonary arterial hypertension treated with nitric oxide. Additionally, there was severe neurological impairment. Considering the unfavorable prognosis and hemodynamic instability, surgical intervention was considered impractical, resulting in the discontinuation of intensive care treatment [8]. In the described cases, the question arises as to whether earlier consideration of splenic hemorrhage could have prevented severe complications or, more critically, a fatal outcome.

Currently, conservative management is the primary treatment for (blunt) splenic injuries in children. With conservative management splenic function might be preserved [2, 3, 11, 12]. Recent data also indicate that the degree of cardiac insufficiency and, in particular, response to fluid resuscitation determine whether surgery is necessary [2, 11]. Given the eventual favorable response observed after fluid resuscitation, the subcapsular splenic hemorrhage was managed conservatively in the second case. In case of an ongoing splenic hemorrhage with persistent cardiorespiratory insufficiency, surgical therapy would have been indicated, which considering the patient’s weight and age, would have meant splenectomy rather than embolization.

This report elucidates the potential implications of neonatal splenic hemorrhage leading to acute and severe cardiorespiratory failure. In both cases, extensive diagnostics were conducted, and empirical treatment initiated prior to the final diagnosis. Notably, splenic hemorrhage was not initially considered in the differential diagnosis by the attending pediatricians or neonatologists, underscoring the challenge in early identification and treatment. Given the rarity of the condition, these two cases provide relevant new insights into the clinical aspects and outcomes of splenic hemorrhage. As splenic hemorrhage can present in various ways, we underscore the significance of incorporating splenic hemorrhage as a pertinent consideration among potential diagnoses for neonatal acute cardiorespiratory failure.

## Data Availability

No datasets were generated or analysed during the current study.

## Abbreviations

CRP: C-reactive protein
CT: Computed tomography
Hb: Hemoglobin
kg: Kilogram
mcg: Microgram
mg: Milligram
MRI: Magnetic resonance imaging
NEC: Necrotizing enterocolitis

## References

[1] Gomella TL, Eyal FG, Bany-Mohammed F. Gomella’s Neonatology: Management, Procedures, On-Call Problems, Diseases, and Drugs, 8e. New York, NY: McGraw-Hill Education; 2020.

[2] De Jong WJ, Nellensteijn DR, Ten Duis HJ, Albers MJ, Moumni ME, Hulscher JB. Blunt splenic trauma in children: are we too careful? Eur J Pediatr Surg. 2011;21(4):234–7. 21404168 10.1055/s-0031-1273692

[3] Kirkegård J, Avlund TH, Amanavicius N, Mortensen FV, Kissmeyer-Nielsen P. Non-operative management of blunt splenic injuries in a paediatric population: a 12-year experience. Dan Med J. 2015;62(2):A4998. 25634501

[4] Chang HP, Fu RH, Lin JJ, Chiang MC. Prognostic Factors and Clinical Features of Neonatal Splenic Rupture/Hemorrhage: Two Cases Reports and Literature Review. Front Pediatr. 2021;9: 616247. 33569364 10.3389/fped.2021.616247 PMC7868555

[5] Eraklis AJ. Abdominal injury related to the trauma of birth. Pediatrics. 1967;39(3):421–4. 6018973 10.1542/peds.39.3.421

[6] Hui CM, Tsui KY. Splenic rupture in a newborn. J Pediatr Surg. 2002;37(4):E3. 11912538 10.1053/jpsu.2002.31641

[7] Perdomo Y, Fiore N, Reyna T. Splenic injury presenting with isolated scrotal findings in a stable newborn. J Pediatr Surg. 2003;38(11):1673–5. 14614724 10.1016/S0022-3468(03)00581-5

[8] Descamps CS, Cneude F, Hays S, Rayet I, Piolat C, Epiard C, Debillon T. Early hypovolemic shock and abdominal distention due to neonatal splenic rupture: urgency of diagnosis and management. Eur J Pediatr. 2017;176(9):1245–50. 28785796 10.1007/s00431-017-2968-y

[9] Tiboni S, Abdulmajid U, Pooboni S, Wighton C, Eradi B, Dagash H. Spontaneous Splenic Hemorrhage in the Newborn. European J Pediatr Surg Rep. 2015;3(2):71–3. 26788451 10.1055/s-0035-1564610 PMC4712061

[10] Ting JY, Lam BC, Ngai CS, Leung WC, Chan KL. Splenic rupture in a premature neonate. Hong Kong Med J. 2006;12(1):68–70. 16495593

[11] Ardley R, Carone L, Smith S, Spreadborough S, Davies P, Brooks A. Blunt splenic injury in children: haemodynamic status key to guiding management, a 5-year review of practice in a UK major trauma centre. Eur J Trauma Emerg Surg. 2019;45(5):791–9. 30251151 10.1007/s00068-018-1014-8

[12] Yung N, Solomon D, Schuster K, Christison-Lagay E. Closing the gap in care of blunt solid organ injury in children. J Trauma Acute Care Surg. 2020;89(5):894–9. 32345899 10.1097/TA.0000000000002757

